# 

**Published:** 1914-05-16

**Authors:** 


### A Lady of Grace of St. John Order of Mercy.

Eleanor Amelia Parkinson, Marchioness Cassar de Sain, of Dover Street, Piccadilly, a Lady of Grace of the Order of St. John Order of Mercy, has left estate valued at £9,855.

### Nottingham Children's Hospital.

Mr. A. M. Williams, who succeeded the late Mr. A. F. Kirby as secretary to the Children's Hospital, Nottingham, is being kept busy over the considerable alterations which are now being carried out to the out-patient department. An operating-room, an *x*-ray installation, and a rest-room are being added, the plans for which have been prepared by Mr. Robert Evans. The new building is expected to cost £2,500.

### Dr. McCulloch and "The Hospital."

In a recent issue of The Hospital (May 2, p. 136) we thought it incumbent to reply to an attack made upon us in the columns of the *Medical Press* by Dr. H. D. McCulloch. In a subsequent issue of the same journal he replies to our article. We do not think his latest addition to the controversy helps him at all to establish his case; and we decline in any circumstances to be drawn into an altercation with him. Our sole reason for publishing this, our last, contribution to the discussion is that of two letters sent us from medical men about a case, we attributed to one a relationship to the patient which it appears was really possessed by the other. This error does not affect in any way the rights or wrongs of Dr. McCulloch's case; but inasmuch as the mistake was made, it is only right that it should be acknowledged and corrected.

### Gifts and Bequests.

Dr. Charles Bruce, LL.D., of Edinburgh, retired banker, who founded and endowed the Bruce Lectureship in Banking at Edinburgh University, has left £1,000 each to the Royal Infirmary, Edinburgh, and the Royal Edinburgh Hospital for Incurables; £750 each to the Edinburgh Institution for the Deaf and Dumb and the Royal Society for Home Relief for Incurables, Edinburgh; and £250 each to twenty other charitable or religious institutions. Subject to certain life interests, he left the following further public bequests: £2,500 to the Edinburgh Royal Infirmary and £2,500 to the Royal Edinburgh Hospital for Incurables.

### Result of the Wireless Appeal.

As the result of the appeal by wireless telegraphy on behalf of the blind, which was referred to in a recent number of The Hospital, the sum of £22 10s. has been obtained, and Commander Wootton, R.N., of the *Royal Edward*, has received from Mr. C. Arthur Pearson a letter thanking him for the generous response to "the wireless call for the blind," in which he says he wishes it had been possible for him to thank all concerned.

### A Hospital Dog Collector.

"Sailor," writes a correspondent, is a clever fox-terrier who has collected upwards of £60 for the Bristol Infirmary. His owner, Miss D. Pullin, of Staple Hill, Bristol, was able to purchase, and fully to equip, a baby's cot out of the money collected by him during 1913. He is six years old, and the possessor of three silver medals, an enamel and bronze cross, and a silver collar, presented at different times by the infirmary in recognition of his usefulness.

### Mr. H. Samuel and Infant Mortality.

The Right Hon. Herbert Samuel, President of the Local Government Board, will give the inaugural address at the National Conference on Infant Mortality, which is to be held at Liverpool on July 2 and 3. Among the subjects down for discussion are milk sterilisation, ante-natal hygiene, the teaching of infant hygiene to the elder girls in elementary schools, the scope and functions of schools for mothers, and the special responsibilities of sanitary authorities in regard to infant welfare.

RATES OF SUBSCRIPTION: Twelve months, 6/6; Six months, 4/-; Three months, 2/-, post free to any address in the United Kingdom. Abroad, Twelve months, 8/8; Six months, 5/-; Three months, 2/6, post free.—Address The Subscription Dept., "THE HOSPITAL," The Hospital Building, 28/29 Southampton Street Strand, London, W.C.

